# Making waves round a structured cloak: lattices, negative refraction and fringes

**DOI:** 10.1098/rspa.2013.0218

**Published:** 2013-09-08

**Authors:** D. J. Colquitt, I. S. Jones, N. V. Movchan, A. B. Movchan, M. Brun, R. C. McPhedran

**Affiliations:** 1 Department of Mathematical Sciences, University of Liverpool, Liverpool L69 3BX, UK; 2 School of Engineering, John Moores University, Liverpool L3 3AF, UK; 3 Dipartimento di Ingegneria Meccanica, Chimica e dei Materiali, Universitá di Cagliari, Piazza d'Armi, 09123 Cagliari, Italy; 4 CUDOS, School of Physics, University of Sydney, New South Wales 2006, Australia

**Keywords:** cloaking, Helmholtz equation, metamaterial lattice, Young's double slit experiment

## Abstract

Using the framework of transformation optics, this paper presents a detailed analysis of a non-singular square cloak for acoustic, out-of-plane shear elastic and electromagnetic waves. Analysis of wave propagation through the cloak is presented and accompanied by numerical illustrations. The efficacy of the regularized cloak is demonstrated and an objective numerical measure of the quality of the cloaking effect is provided. It is demonstrated that the cloaking effect persists over a wide range of frequencies. As a demonstration of the effectiveness of the regularized cloak, a Young's double slit experiment is presented. The stability of the interference pattern is examined when a cloaked and uncloaked obstacle are successively placed in front of one of the apertures. This novel link with a well-known quantum mechanical experiment provides an additional method through which the quality of cloaks may be examined. In the second half of the paper, it is shown that an approximate cloak may be constructed using a discrete lattice structure. The efficiency of the approximate lattice cloak is analysed and a series of illustrative simulations presented. It is demonstrated that effective cloaking may be obtained by using a relatively simple lattice structure, particularly, in the low-frequency regime.

## 1. Introduction

Following the publication of two seminal papers in 2006 by Pendry *et al.* [1] and Leonhardt [2], there has been very considerable interest in the concept of invisibility cloaks and many scholarly works have been devoted to it; for example, see the recent review article Guenneau *et al*. [3] and references therein. The experimental validation of cloaking by Schurig *et al.* [4] has further increased both scholarly, and popular, interest in invisibility cloaks. The concept of cloaking via transformation optics is due to an earlier fundamental result by Greenleaf *et al.* [5,6] on singular transformations and applications to cloaking for conductivity. The fundamental property that enables objects to be cloaked from electromagnetic waves is the metric invariance of Maxwell's equations [7,8].

The classical approach to cloaking via transformation geometry involves deforming a region such that a point is mapped to a finite region corresponding to the inner boundary of the cloak. Usually, such transformations involve transforming a point into a finite region with a smooth boundary, such as an ellipse [1,9,10]. The mapping is non-singular everywhere, except at the initial point which is deformed into the inner boundary of the cloak. Since the material properties of the cloak are determined by the transformation [11], such singular transformations lead to material properties that are singular. Greenleaf *et al.* [12,13] addressed this issue in two papers and derived the condition for finite energy solutions for weak solutions of the Helmholtz equation and Maxwell's equations. In [12], it was demonstrated that finite energy solutions to the cloaking problem for the Helmholtz equation exist for an object with a single layer cloak. However, for the case of Maxwell's equations with internal currents, the cloaking of an infinite cylinder cannot be achieved with a single layer or without imposing a physical surface at the inner boundary of the cloak. In the same paper, Greenleaf *et al.* derived an identity linking the transformed scalar wave equation to the metric of the deformed space, which may then by linked to the material properties of the cloak [13]. In 2008, Norris [9] studied acoustic cloaking and re-derived an equivalent identity to that in [12] using the framework of finite elasticity, leading to a cloak with a density described by a rank 2 tensor. Moreover, it was demonstrated that the total mass of the cloak is infinite for the case of perfect cloaking. Norris further demonstrated that the problem of infinite mass could be overcome if both the density and elastic properties of the cloak were anisotropic. An alternative approach to negate the problem of singular material properties is to construct a so-called *near cloak* by regularizing the transformation [14]. Rather than mapping a single point to the inner boundary of the cloak Kohn *et al.* [14] proposed mapping a ball of small, but finite, radius to the inner boundary. A small regularization parameter which characterizes the initial radius of the ball is introduced, which results in a non-singular mapping on the cloak and its boundary. The regularization procedure was used to create illustrative *near cloaks* in [9].

In 2006, Milton *et al.* [15] examined how the equations of motion for a general elastic medium transform under an arbitrary curvilinear transformation. It was shown that *a priori* requiring a symmetric stress tensor enforces a particular choice of the gauge (i.e. the manner in which the displacement transforms). It was found that, in general, the equations of motion are not invariant under transformation but are mapped to a more general system with non-scalar density. In the same paper, Milton & Willis [16] demonstrated that a special case of the so-called Willis equations [16] remain invariant under general curvilinear transformations. In [15], identities linking the material properties of the transformed material to the transformation, for both classical elasticity and the more general Willis materials, are derived. In 2011, Norris & Shuvalov [17] further generalized the work of Milton *et al.*, deriving a more general system of transformed equations without imposing the constraint of symmetric stress. The material properties of the transformed system were derived explicitly and shown to depend on both the transformation and choice of gauge. Together [15,17] provide a comprehensive framework in which to investigate cloaking in elastodynamics.

A design for a cloak to control flexural waves in thin plates was proposed by Farhat *et al.* [18]. The cloak is constructed by several concentric layers of piecewise constant isotropic elastic material. Farhat *et al.* also presented a simplified model suitable for practical implementation with 10 layers using six different materials. Following Farhat *et al.* [18], an experimental group led by Wegener fabricated a cloak using 20 concentric rings and 16 different elastic metamaterials [19]. Physical measurements were compared with numerical simulations and found to be in good agreement. Control of in-plane waves governed by the Navier equations was examined by Brun *et al.* [20]. In [20], the authors modelled a circular cloak using the classical radial transformation by deforming a disc to an annulus. The efficiency of the cloak was illustrated using finite element simulations and the numerical solution of the cloaking problem was compared with the Green's function for a homogeneous elastic space.

An influential paper of Rahm *et al.* [21] presents a transformation optics algorithm for a problem of electromagnetism involving a cloak of a square shape. The transformation is performed in Cartesian coordinates and results in a piecewise smooth cloak on the interior points, with matching regions in the neighbourhood of corners and a singularity at the origin transformed into the inner boundary of the cloak. The model of such a continuum cloak received substantial attention and subsequent use by the modelling community [22–27]. In the majority of these papers, the emphasis is on the geometrical aspect of the possible shapes of the cloak, with examples ranging from polygonal and elliptical cloaks to heart-shaped cloaks. Although it is indeed interesting to see a wide range of transformations and geometries, it also remains important to understand the transformed problem in the context of the physical model, address the analysis of the transformed boundary or transmission conditions and furthermore derive the properties of the solutions. The paper [21], which stimulated a good level of discussion on the topic, also admits a deficiency regarding the analysis of the solution near the boundary of the cloak. Apparently, no indication is given about the sensitivity of the result to the type of boundary conditions (Dirichlet or Neumann) on the inner boundary of the cloak. The authors' evaluation of the effectiveness of the cloaking is based on a visual observation linked to a numerical simulation at a single frequency. On p. 91, the text of [21] says ‘Although the effective *μ*_*x*_ is inaccurate in the vicinity of the boundary of the inner square, it can be shown, that the performance of the implemented device is not affected by this fact, which is out of the scope of this paper’. Indeed, if the authors had attempted to change the frequency range, they would have seen significant differences. Hence, the cloak advocated in [21] is an approximate cloak, where the boundary effects become important and visible as the frequency of the incident waves increases.

The ideas of metric invariance in Maxwell's equations and cloaking have been taken on board as a technical tool and on many occasions, the researchers omit to look at the physical model corresponding to the transformed equations. For example, on p. 99 in [25] the text reads ‘The square cloak has the same geometry as the cylindrical case, except that we replace the cylindrical shell by a rectangular shell with the same size’. This comparison of unlike geometries omits important effects, such as field concentrations near sharp corners, which make cloaking more difficult. Motivated by Rahm *et al*. [21], Farhat *et al.* [22] attempted to construct an approximate square cloak for out-of-plane shear waves. Farhat *et al.*, using the method of multiple scales, introduced a microstructure composed of a regular array of perforations and derived a homogenized continuum which would approximate an ideal cloak. However, as Farhat *et al.* admit on p. 15 of [22] ‘it is fair to say that our structured cloak is not as efficient as we would have expected’.

Polygonal cloaks have also been the subject of experimental investigation. For example, Chen & Zheng [28] report the results of an experimental broadband hexagonal cloak based on a piecewise linear homogeneous transformation. Although the cloak does not render the cloaked object invisible, it does reduce its apparent size. The cloak is demonstrated to work for visible light. However, Chen & Zheng emphasize that the cloak only functions for light incident from six directions defined by the faces of the hexagon. More recently, Landy & Smith [29] produced an experimental unidirectional metamaterial cloak for microwaves. The reported cloak [29] is based on a bilinear transformation, which maps a line segment to a two-dimensional region of space. Cloaks based on such transformations are referred to as *carpet cloaks* in the literature [30]. The advantage of such cloaks is that the requisite material properties are homogeneous and finite. However, Landy & Smith [29] admit that such cloaks are only effective over a narrow range of observation angles. The cloak is nonetheless impressive given that the practical implementation does not rely on the eikonal approximations as is the case with other implementations [31–33].

It appears that the work reported in [21] has generated a scope for further discussion and indeed further improvement of the model involving ‘glued transforms’ that lead to approximate rectangular cloaks. Such cloaks are by no means exact and are frequency-sensitive. A regularization procedure, as illustrated by Kohn *et al.* for the spherical cloak [14], can be applied to make the transformation, and hence the material properties, non-singular on the inner boundary of the cloak. The regularization procedure not only simplifies the analysis, but also makes it physically meaningful. Furthermore, a lattice approximation is straightforward for a regularized square-shaped cloak. This appears to be efficient and serves a relatively wide frequency range.

It should be emphasized that the current paper improves significantly the influential work by Rahm *et al.* [21]. In particular, the square *push out* transformation is regularized and it is demonstrated that this construction of a *near cloak* via regularization does not adversely affect the effectiveness of the cloaking. Detailed ray analysis is carried out and the ray equations are derived. An objective numerical measure of the quality of the cloaking is introduced and used to quantify the effects of frequency, interface conditions and orientation of the inclusion. Illustrative simulations are produced for cylindrical sources and the analytical ray diagrams presented. For physical fabrication, a discrete lattice cloak is used to approximate the regularized continuum cloak and it is shown that the lattice structure acts as an effective cloak, particularly at low frequencies.

In this paper, the emphasis is on solutions of the Helmholtz equation. The Helmholtz equation arises in a wide variety of fields including electromagnetism, elasticity and acoustics. Therefore, solutions to the cloaking problem for the Helmholtz equation have a wide range of potential applications. However, for definiteness and ease of exposition the language of elasticity will be used throughout this paper.

**Figure 1.**
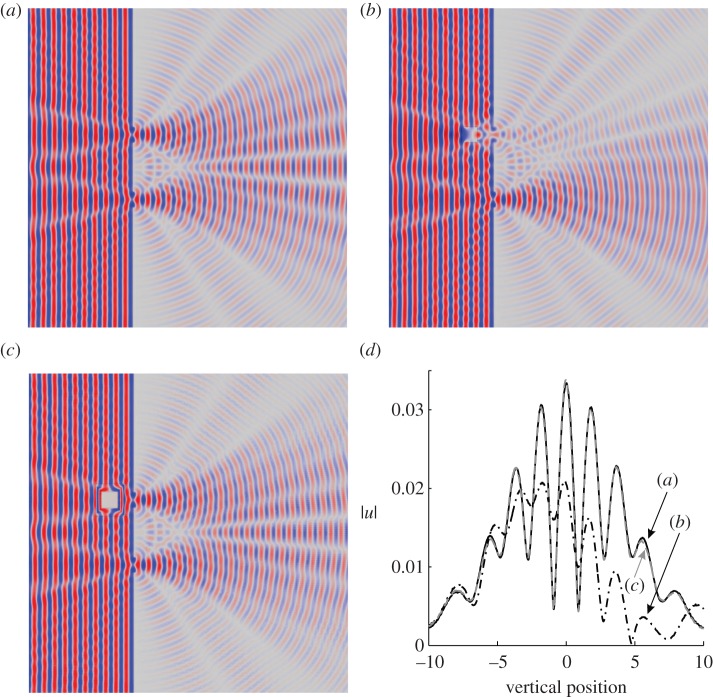
(*a*–*c*) The displacement amplitude field *u*(***x***) for the Young's double slit experiment with no inclusion, an uncloaked inclusion and a cloaked inclusion, respectively. (*d*) A plot of |*u*(***x***)| over the observation screen illustrating the interference fringes for cases (*a*–*c*). (An animated version of this figure may be found in the supplementary material.) (Online version in colour.)

The current paper is structured as follows. A description of the regularized cloak in the continuum model of out-of-plane shear elastic waves follows the introduction. This also includes the discussion of the essential and natural interface conditions on the boundaries of the cloak. An explicit ray tracing algorithm is developed, and the phenomenon of negative refraction on the interface boundaries is explained. Numerical scattering measures are included, with detailed simulations. The analysis also incorporates Neumann and Dirichlet boundary conditions on the inner contour of the cloak. As a demonstration of the effectiveness of the regularized cloak, a Young's double slit experiment is presented. A recent paper by Greenleaf *et al.* [34] considers an application of cloaking via transformation optics in quantum mechanics. In particular, Greenleaf *et al.* present a class of invisible reservoirs and amplifiers for waves and particles. The issues discussed below, which are raised by this linkage between cloaking and quantum mechanics, are in some ways similar to those discussed by Greenelaf *et al.* To give the reader an idea of the results which follow, part of a simulation of the interference pattern owing to a plane wave interacting with two apertures is shown in figure 1. The same figure presents the fringe structure for the case of an aperture obstructed by an uncloaked and then cloaked inclusion. This numerical experiment convincingly demonstrates the high quality of the cloaking effect produced by this regularized cloak. Further discussion of the Young's double slit experiment is deferred to §3. It is also shown that one of the undeniable advantages of such an approximate cloak is the straightforward connection with the discrete lattice structures. These connections are analysed in detail, and accompanied by a range of physical simulations. Concluding discussion and a final outline are included in the last section of this paper.

## 2. The regularized continuum cloak

**Figure 2.**
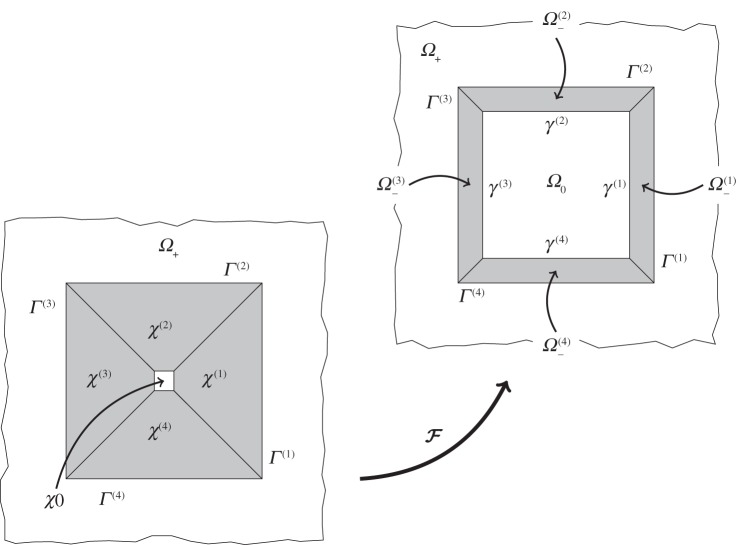
The transformation 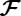 maps the undeformed region *χ* to the deformed configuration *Ω*_−_. The boundary between *Ω*_+_ and *Ω*^(*i*)^_−_ is denoted *Γ*^(*i*)^, while the interface between *Ω*_0_ and *Ω*^(*i*)^_−_ is denoted *γ*^(*i*)^. The corresponding boundaries in the undeformed configuration are denoted by *Γ*^(*i*)^ and *σ*^(*i*)^, respectively.

The classical approach to cloaking via transformation geometry involves deforming a region such that a point is mapped to a finite region corresponding to the inner boundary of the cloak. Indeed, the square *push out* transformation proposed by Rahm *et al.* [21] maps a point to a square. The mapping is non-singular everywhere except at the inner boundary of the cloak. In the present paper, a regularized version of the square *push out* transformation is used. In particular, the trapezoids *χ*^(*i*)^ are mapped to the trapezoids *Ω*^(*i*)^_−_ as illustrated in figure 2 with continuity, but not smoothness, imposed on the interfaces between the four trapezoids. The mapping is non-singular on the closure of the cloak, and, hence, all corresponding material properties are finite. It will be shown that this regularized transformation yields an effective broadband cloak, with finite material properties which may easily be approximated by a regular lattice.

### (a) The transformation

Consider a small square 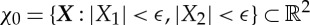, which via the map 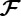 is mapped to the square 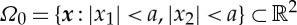. Physically, *w* is the thickness of the cloak, *a* is the semi-width of the inclusion *Ω*_0_ and *ϵ* is the initial semi-width of the square *χ*_0_ where 0<*ϵ*/*a*≪1. In this case, it is convenient to decompose the cloak into four sub-domains *χ*=*χ*^(1)^∪⋯∪*χ*^(4)^, as illustrated in figure 2. Formally, 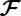 defines a pointwise map from ***X***∈*χ*=*χ*^(1)^∪⋯∪*χ*^(4)^ to 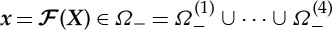. The mapping is continuous and nonlinear on *χ*, and defined in a piecewise fashion such that 
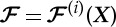
 for ***X***∈*χ*^(*i*)^, where

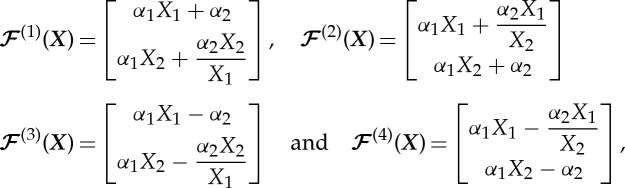
with *α*_1_=*w*/(*a*+*w*−*ϵ*) and *α*_2_=(*a*+*w*)(*a*−*ϵ*)/(*a*+*w*−*ϵ*). The exterior of the cloak remains unchanged by the map, that is, 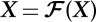 for 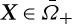, where the bar denotes the closure of the domain. The Jacobian matrices and determinants are then

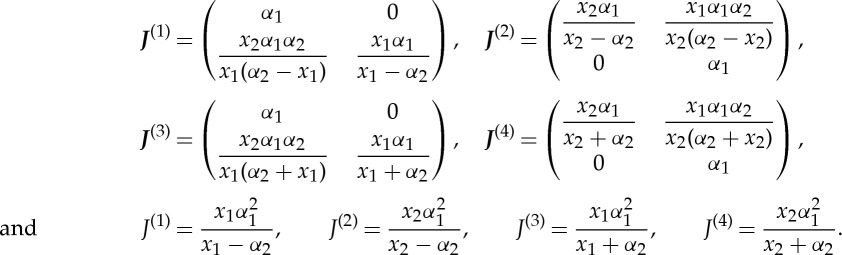
It is emphasized that 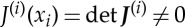 for 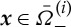 and *ϵ*≠0, that is, the map is continuous on both the interior and boundary of the cloak. The metric of the deformed space 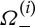 is ***g***^(*i*)^=(***J***^(*i*)^***J***^(*i*)^^T^)^−1^.

The present paper will be devoted to the propagation of time harmonic out-of-plane shear waves of radian frequency *ω* and displacement amplitude *u*(***x***). Lemma 2.1 in [9] allows the Helmholtz equation for an isotropic homogeneous medium *μ*∇_***X***_⋅(∇_***X***_)*u*(***X***)+*ϱω*^2^*u*(***X***)=0 for ***X***∈*χ* to be written in deformed co-ordinates as
2.1
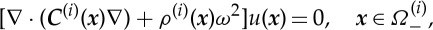
where *μ* is the constant ambient stiffness, *ϱ* is the constant ambient density, ***C***^(*i*)^(***x***)=[*μ*/*J*^(*i*)^(***x***)]***J***^(*i*)^(***x***)[***J***^(*i*)^(***x***)]^T^ is the transformed stiffness matrix and *ρ*^(*i*)^(***x***)=*ϱ*/*J*^(*i*)^(***x***) is the transformed density. The differential operator ∇_***X***_ is written in the undeformed space and should be distinguished from ∇ which is written in the deformed coordinates.

Since the mapping is continuous on 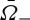, the material properties of the cloak are non-singular. The transformed stiffness tensor is symmetric and positive-definite. Physically, the transformed material properties correspond to a heterogeneous anisotropic medium.

### (b) Interface conditions

Without loss of generality, it is convenient to restrict the following analysis to a single side of the cloak. With reference to figure 2, consider the sub-domain 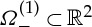 in the absence of the inclusion and remaining three sides of the cloak. In the absence of sources the out-of-plane shear deformation amplitude of an outgoing time-harmonic wave of angular frequency *ω* satisfies the following equation:
2.2
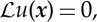
together with the Sommerfeld radiation condition at infinity. Here, 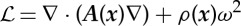 is the Helmholtz operator, ***A***(***x***) and *ρ*(***x***) are defined as
2.3
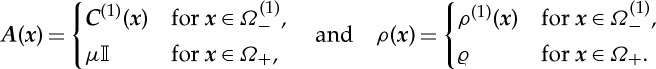


Let *v*(***x***) be a continuous piecewise smooth solution of the Helmholtz equation in 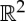 satisfying the Sommerfeld radiation condition at infinity. Integrating the difference 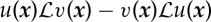 over a disc 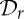 of radius *r* containing 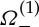 yields

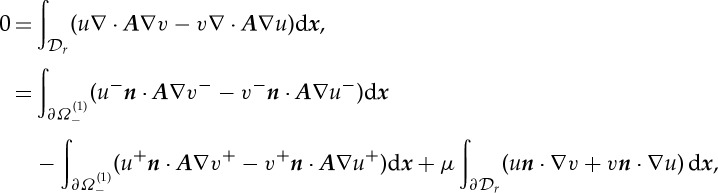
where the fact that ∇*u*⋅***A***∇*v*=∇*v*⋅***A***∇*u* (since ***A*** is symmetric) has already been used. Since *u*(***x***) and *v*(***x***) represent outgoing solutions, the final integral vanishes as 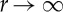. Thus, the essential interface condition is continuity of the field
2.4
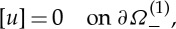
and the natural interface condition is continuity of tractions
2.5
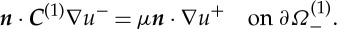
These interface conditions are equivalent to those derived in [9] for acoustic pressure by application of Nanson's formula and imposing particle continuity.

### (c) The cloaking problem

Consider the propagation of time harmonic out-of-plane deformations, generated by a point source, in a homogeneous infinite elastic solid in which is embedded an inclusion surrounded by a cloak. The displacement amplitude field then satisfies
2.6
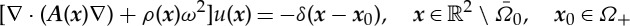
and
2.7
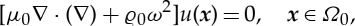
with continuity of *u*(***x***) and tractions on all internal boundaries according to (2.4) and (2.5). Additionally, the Sommerfeld radiation condition is imposed at infinity. The stiffness tensor ***A***(***x***) and density *ρ*(***x***) are
2.8
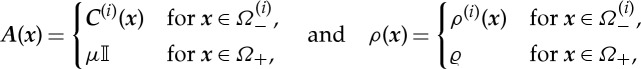
and *μ*_0_ and *ϱ*_0_ are the stiffness and density of the inclusion, respectively.

### (d) The ray equations

While, in principle, the exact wave behaviour of the displacement field can be obtained from the solution of the cloaking problem, it is illuminating to consider the leading order behaviour of rays through the cloak. Consider a WKB expansion [35,36] of the displacement amplitude field in terms of angular frequency *ω*, and the amplitude and phase functions *U*_*n*_(***x***) and *φ*(***x***), respectively,
2.9
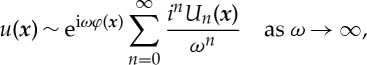
whence the leading order equation for the phase on the interior of the cloak is
2.10
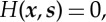
where *H*(***x***,***s***)=*μϱ*^−1^***s***⋅***g***^−1^***s***−1, ***s***=∇*φ* is the slowness vector, *μ* and *ϱ* are the stiffness and density of the ambient medium, respectively, and ***g*** is the metric of the transformation. In terms of wave propagation, the conserved quantity *H*(***x***,***s***) represents the first-order slowness contours [37]. The characteristics of the quantity *H*(***x***,***s***) then satisfy the following system:
2.11
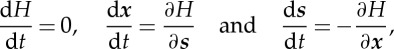
where *t* is the ray parameter (equivalently, the time-like parameter). At this point, it is convenient to introduce index summation notation where summation, from 1 to 2, over repeated indices is implied. The system (2.11) may then be expressed as
2.12
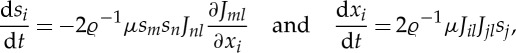
where *J*_*ij*_=(***J***)_*ij*_ are the components of the Jacobian matrix and should be distinguished from the *J*, the Jacobian determinant. The superscript labels have been omitted for brevity, but *J*_*ij*_ and *J* should be understood as 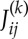 and *J*^(*k*)^ for *k*=1,…,4 corresponding to the four sides of the cloak.

From equation (2.10), an alternative representation, in terms of wave normals ***n*** and the phase velocity *v*, is
2.13
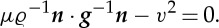
The representation (2.13) is obtained by assuming a plane wave solution to the Helmholtz equation [37]. Alternatively, seeking a solution of the full wave equation in the form of the leading term in a WKB expansion:

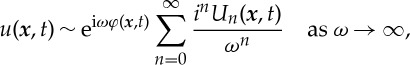
yields the same result with ∂*φ*/∂*t*=*v*. From (2.10) and (2.13), the slowness vector can be expressed in terms of the original material properties (through *ϱ* and *μ*) and the map (through ***J***) as
2.14
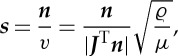
Further, in the undeformed configuration, the equivalent conserved quantities are *μϱ*^−1^***S***⋅***S***−1=0 and *μϱ*^−1^=*V* ^2^. Together with (2.10) and (2.13), these two equations imply that
2.15
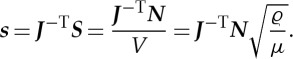


Now, consider a ray (line) in the ambient medium, in direction ***N*** passing through ***X***_0_ and parametrized by *t*. The corresponding curve in the cloak is 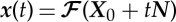, whence

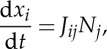
which using (2.15) may be rewritten thus
2.16
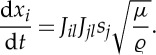
Taking the derivative of (2.15) for constant ***N*** yields

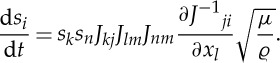
Using the compatibility condition that the deformation gradient should be irrotational under finite deformation *ϵ*_*jk*ℓ_∂*J*^−1^_*ik*_/∂*x*_*j*_=0_ℓ*i*_, the partial derivative above may be written as ∂*J*^−1^_*jl*_/∂*x*_*i*_, whence
2.17
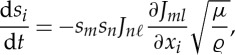
where *ϵ*_*jk*ℓ_ is the permutation tensor and the equality 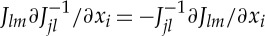 has already been used. Consider the characteristic equations for the waves in the cloak (2.12), together with the equations of the transformed rays (2.16) and (2.17). The system (2.16) and (2.17) are the equations of characteristics in the cloak, up to an arbitrary scaling constant of 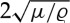. Thus, to leading order, rays (or straight lines) in the ambient medium map directly to rays in the cloak.

**Figure 3.**
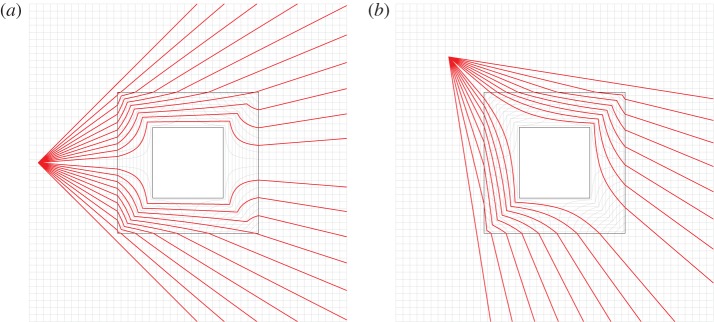
Plots of the ray paths through the cloak for a cylindrical source. The grey lines indicate the deformation of the space inside the cloak. (Animated versions of these figures may be found in the electronic supplementary material.) (Online version in colour.)

Figure 3 shows rays emanating from a point source, passing through the cloak and emerging from the cloak on their original trajectory. In this sense, the object is ‘invisible’ to an observer outside the cloaking region. The figure clearly illustrates how wave propagation in the cloak is related to the map. Animated versions of figure 3 can be found in the electronic supplementary material.

An interesting alternative perspective is apparent if figure 3 is viewed, not as rays diverging from a source, but as rays converging to a focal point. It is observed that the rays converge to the focal point around the inclusion. One can envisage several applications where such an effect may be useful. For example, image distortion from the mirror mounts in telescopes could be reduced by cloaking the mounts. In addition, apparatus and mounting structures on microwave receivers could be cloaked to improve the quality of the signal. One could also conceive of cloaking mounting points and the surrounding structures in laser cutting machines to protect them from accidental damage.

#### (i) Negative refraction

It is evident from figure 3 that while the rays are continuous, they are not necessarily differentiable. In particular, at the interface between the cloak and ambient medium, and at the internal interfaces of the cloak, refraction occurs characterized by a discontinuity of the spatial derivatives of the rays. Of particular interest are the regions on the outer boundary of the cloak where negative refraction occurs.

Consider figure 3*a*. Negative refraction occurs on the right-hand interface between the cloak and the ambient medium. A ray exiting the right-hand side of the cloak with gradient *M* at point ***X***^(0)^=***x***^(0)^ may be described by the equation 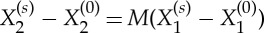 in the ambient medium, where ***X***^(*s*)^ is the position of the source. The behaviour of the ray at the interface is entirely characterized by the position of the source relative to the interface and its initial gradient. Therefore, without loss of generality, the following analysis may be restricted to the right-hand side of the cloak. On the interior of the right-hand side of the cloak, the ray exiting the cloak at ***x***^(0)^ is characterized by

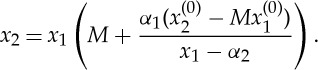
The gradient of the ray as it approaches the exterior boundary from the interior of the cloak is then

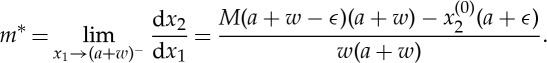
Thus, the gradient is discontinuous at the exterior interface. For negative refraction it is required that m*M<0, which leads to the following inequalities:
2.18
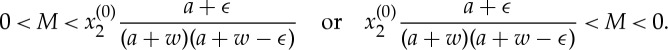
Assuming that the source lies on the line *X*_2_=0 as in figure 3*a* the inequalities reduce to the single inequality

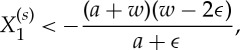
which is satisfied for all sources outside the cloak 
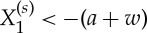
, if *w*<*a*+3*ϵ*. Thus, for a sufficiently thin cloak and a cylindrical source placed along *X*_2_=0 and at any distance from the cloak, negative refraction is expected on the opposite side of cloak.

Alternatively, for a source located along the line *X*_1_=0 the inequalities (2.18) become
2.19
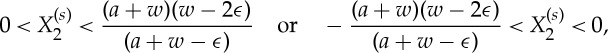
where the fact that 
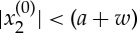
 has been used. Since *a*,*w*>0, and 0<*ϵ*/*a*≪1, the above inequalities are never satisfied. Hence, the lack of negative refraction on the horizontal interfaces in figure 3*a*. Similar arguments may be used to consider other regions where negative refraction may or may not occur. It is observed that negative refraction always occurs at the interface between the four regions of the cloak, where the material properties (equivalently the transformation) are not smooth.

### (e) Scattering measure

It is desirable to have some quantifiable measure of the quality of the cloak with respect to shielding, rather than relying on visual observations. However, it is not obvious what ‘quality’ means with respect to a cloak, given that there are essentially three fields involved, that is, the ideal field in the absence of both cloak and inclusion, the uncloaked field with an inclusion present but without a cloak, and the cloaked field with both the inclusion and cloak. Previous experimental studies [19] have used an *L*_2_ norm computed directly from the measured fields to place a numerical value on the quality of the cloak. It is in this spirit that the following ‘scattering measure’ is formally introduced as a tool to quantify the cloaking effect:
2.20
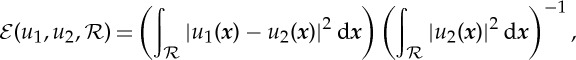
where 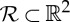 is some region outside the cloak, and *u*_1_(***x***) and *u*_2_(***x***) are any two fields. In the present paper, the quantities 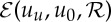 and 
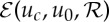
 are presented for a series of illustrative simulations. The field 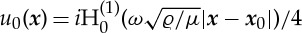 is the Green function for the unperturbed problem and represents the ‘ideal’ field, *u*_*u*_(***x***) and *u*_*c*_(***x***) are the uncloaked and cloaked fields, respectively. Thus, perfect cloaking corresponds to a vanishing 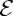. Along with the raw scattering measures an additional quantity, 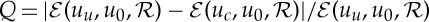, is also presented. The parameter *Q* characterizes the relative reduction of the scattering measure by the introduction of a cloak. It should be emphasized that this is only one of a number of possible measures of quality.

**Figure 4.**
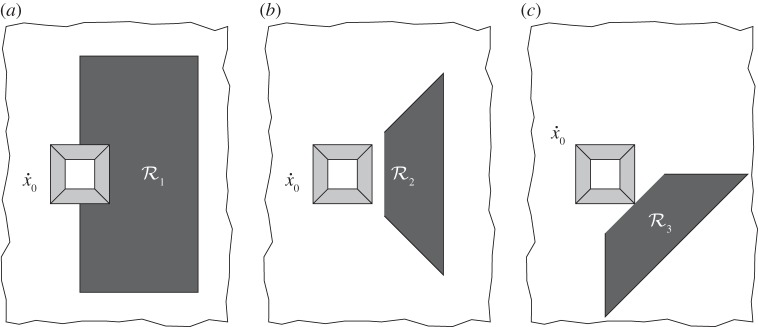
The three regions used for computation of the scattering measure. (*a*) Strict scattering region, (*b*) forward scattering and (*c*) corner scattering.

*Choice of* 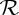. For the purpose of illustration three different regions of integration are considered, as shown in figure 4. The three regions used were chosen as follows: (a) 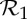 is the most strict region used taking into account significant near-field effects and a wide range of scattering angles. However, it is unlikely that this region would be measurable in practice. (b) The forward scattering region (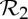) is relevant if the scattered field is measurable over a wide range of forward scattering angles. (c) The corner scattering region (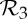) is employed for sources located along the diagonal of the square inclusion. It is emphasized that 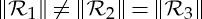, and the leading edges of the regions 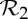 and 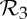 are located at the same distance from the source.

In the following section, the scattering measures will be presented for a series of illustrative simulations.

### (f) Illustrative simulations

**Figure 5.**
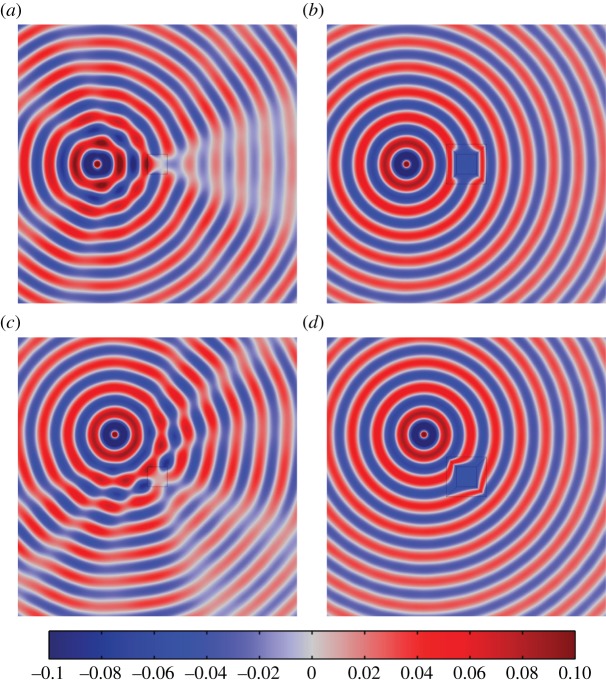
Plots of the field *u* for the uncloaked and cloaked square inclusion, where the angular frequency of excitation is *ω*=5. The position of the source is as follows. (*a*) Uncloaked, *x*_0_=[−3,0]^T^, (*b*) cloaked, *x*_0_=[−3,0]^T^, (*c*) uncloaked, 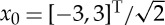 and (*d*) cloaked, 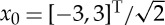. (Online version in colour.)

**Figure 6.**
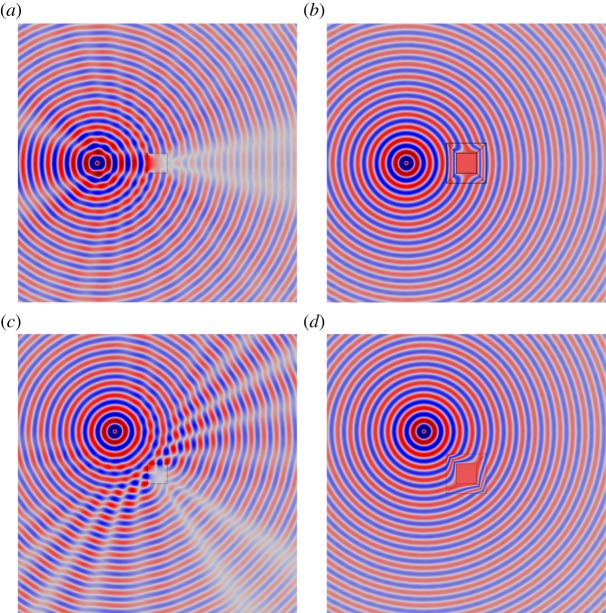
Plots of the field *u* for the uncloaked and cloaked square inclusion where the angular frequency of excitation is *ω*=10. The position of the source is as indicated below and the inclusion is located at the centre of the image in all cases. The colour scale is as indicated in figure 5. (*a*) Uncloaked, *x*_0_=[−3,0]^T^, (*b*) cloaked, *x*_0_=[−3,0]^T^, (*c*) uncloaked, 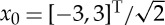 and (*d*) cloaked, 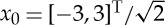. (Online version in colour.)

A series of illustrative simulations were created using the finite element software COMSOL Multiphysics. Perfectly matched layers were used in the vicinity of the boundary of the computational domain in order to simulate an infinite domain. For the purposes of these computations, the following non-dimensional parameter values^1^ were chosen: *a*=0.5, *w*=0.5, *μ*=*ϱ*=1, *μ*_0_=0.1, *ϱ*_0_=0,*ϵ*=1×10^−6^. Figures 5 and 6 show the displacement amplitude field *u*(***x***) for a cylindrical source oscillating at *ω*=5 and 10, respectively. The figures clearly illustrate the efficacy of the square cloak, even at relatively high frequencies. Table 1 shows the corresponding scattering measures as introduced in §2*e*. It is clear that this square ‘push out’ cloak is highly effective. Indeed, for the illustrative simulations presented here, the cloak reduces the scattering measure by not less than 99.62 per cent compared with the uncloaked inclusion.

**Table 1.** The scattering measures corresponding to the simulations shown in figures 5 and 6.

source		scattering measure 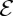		
position	frequency	uncloaked	cloaked	*Q*
*scattering region* 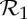				
[−3,0]^T^	5	0.1529	4.351×10^−4^	0.9972
[−3,0]^T^	10	0.1455	4.514×10^−4^	0.9969
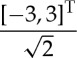	5	0.2002	3.941×10^−4^	0.9980
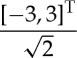	10	0.3286	4.068×10^−4^	0.9988
*scattering region* 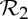				
[−3,0]^T^	5	0.3224	3.664×10^−4^	0.9989
[−3,0]^T^	10	0.3093	1.167×10^−3^	0.9962
*scattering region* 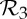				
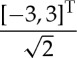	5	0.2988	3.654×10^−4^	0.9988
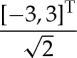	10	0.2988	7.803×10^−4^	0.9974

**Figure 7.**
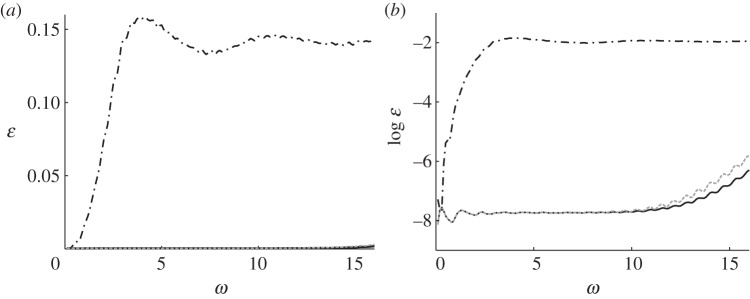
(*a*) The figure shows the scattering measure plotted against angular frequency. (*b*) The figure shows the log of the scattering measure plotted against angular frequency. The solid line corresponds to the continuum in the absence of both an inclusion and cloak. The dashed line represents the cloaked inclusion and the dash-dot line corresponds to the uncloaked inclusion. The region 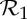 (see figure 4 and the associated text) was used to compute the error measure.

Figure 7 shows the scattering measure plotted against non-dimensional angular frequency *ω* (with *μ*=*ϱ*=1). The solid curve in figure 7 corresponds to the continuum, in the absence of both cloak and inclusion. This curve gives an indication of the numerical error in the simulation induced by, for example, the use of perfectly matched layers and the numerical discretization. The dashed curve corresponds to the cloaked inclusion, while the dash-dot curve corresponds to the uncloaked inclusion. It is observed that the numerical measure of the cloaked inclusion remains close to that of the intact continuum for a large range of frequencies. Moving to dimensional quantities, suppose the simulation corresponded to a particular polarization of an electric wave travelling through glass at a speed of approximately 2×10^8^ m s^−1^. The line *ω*=10 in figure 7 then corresponds to a frequency of approximately 340 MHz.

#### (ii) Boundary considerations

**Figure 8.**
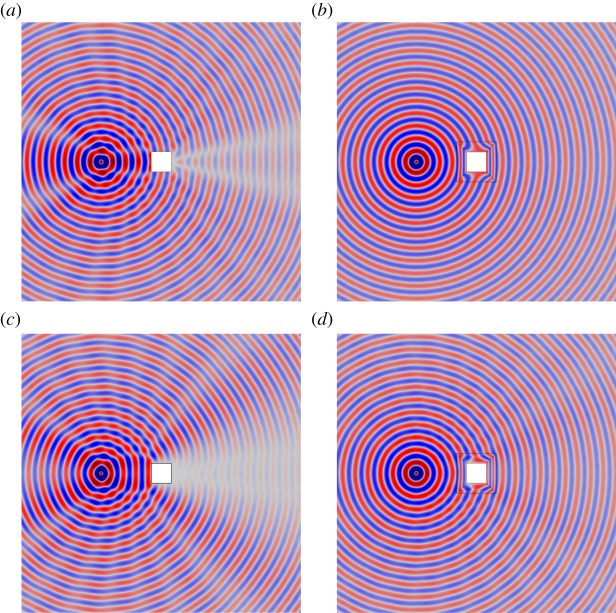
Plots of the field *u* for the uncloaked and cloaked square inclusion with Neumann boundary conditions in (*a*,*b*), and Dirichlet boundary conditions in (*c*,*d*). Here, the source is located at *x*=[−3,0]^T^ and oscillates at *ω*=10. The colour scale is as indicated in figure 5. (*a*) Uncloaked, Neumann, (*b*) cloaked, Neumann, (*c*) uncloaked, Dirichlet and (*d*) cloaked, Dirichlet. (Online version in colour.)

While cloaking via transformation geometry has been extensively treated in the literature, the sensitivity of the cloaking effect to the boundary conditions is rarely discussed. The cloak is formed by deforming a small region (a point in the case of the classical radial transformation [1]), into a larger finite region. If the region is an inclusion, then the natural interface conditions may be determined following the method outlined in §2*b*. If the cloaked region is a void or rigid inclusion, however, there is some freedom in choosing the boundary condition, subject to the constraints of the physical problem. Figure 8 shows the field *u*(***x***) for a cloaked void, with Neumann (parts (*a*) and (*b*)) and Dirichlet (parts (*c*) and (*d*)) conditions applied to the interior of the cloaked region. The corresponding scattering measures are shown in table 2.

**Table 2.** The scattering measures for a void with Neumann and Dirichlet boundary conditions. Here the source is located at [−3,0]^T^.

source		scattering measure 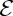		
boundary condition	frequency	uncloaked	cloaked	*Q*
*scattering region 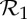 *				
Neumann	5	0.1624	4.351 × 10^−4^	0.9973
Neumann	10	0.1558	4.540 × 10^−4^	0.9971
Dirichlet	5	0.2931	1.038 × 10^−2^	0.9646
Dirichlet	10	0.2553	7.875 × 10^−3^	0.9692
*scattering region 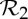 *				
Neumann	5	0.3436	3.664 × 10^−4^	0.9989
Neumann	10	0.3258	1.163 × 10^−3^	0.9964
Dirichlet	5	0.4864	1.566 × 10^−2^	0.9678
Dirichlet	10	0.5030	1.673 × 10^−2^	0.9667

Although the square cloak is effective in both cases, it is clear from both the figures and the table of scattering measures that the type of boundary condition imposed on the cloaked object affects the quality of the cloaking. Indeed, for a void (Neumann) the cloaking reduces the scattering measure by between 99.7 and 99.9 per cent for both *ω*=5 and 10. In contrast, cloaking reduces the scattering measure of a rigid inclusion (Dirichlet) by between 96.5 and 96.8 per cent for *ω*=5 and between 96.7 and 96.9 per cent for *ω*=10. The effect of the boundary condition may be interpreted in the following way. As a result of the transformation, the cloaked object and cloak together behave as if the void is small. In this sense, the cloaked inclusion represents a singular perturbation of the fundamental solution of the Helmholtz equation. In the case of a free void with Neumann conditions, the leading order term in the asymptotic expansion is the dipole term, which is of order *ϵ*^2^ and decays like the first derivative of the fundamental solution. On the other hand, for a fixed void with Dirichlet conditions, the leading order term in the expansion is the monopole term which is of order *ϵ* and decays like the fundamental solution. Thus, the perturbation from the free void is smaller than the perturbation from the fixed void, leading to improved cloaking.

## 3. Cloaking path information

In recent years, there has been much interest in experiments to elucidate the fundamental principles of quantum mechanics, and, in particular, the relationship between measurement and system behaviour. One basic experiment with its variant features in many such experimental studies is the classical Young's double slit experiment (e.g. [38]). This suggested that it may be of interest to consider the interaction of the excellent mechanical cloaking demonstrated earlier with the foundational quantum mechanics experiment.

Thus, a Young's double slit experiment is considered where a monochromatic plane wave is incident on a screen with two apertures. Owing to the superposition of the waves passing through the two apertures, the distinctive double slit interference pattern is produced on an observation screen placed on the opposite side of the apertures to the source. The result of a simulation of the standard experiment is shown in figure 1*a*, with the diffraction pattern produced on the observation screen (in this case, a vertical line near the right-hand edge of figures 1*a*–*c*) shown as curve (*a*) in figure 1*d*. Placing an object (inclusion) over one slit, as in figure 1*b*, partially destroys the diffraction pattern. The corresponding pattern on the observation screen is shown as line (*b*) in figure 1*d*. However, coating the object with the square *push out* cloak presented earlier, as shown in figure 1*c*, restores the original diffraction pattern almost entirely. The interference pattern corresponding to the cloaked object is shown as curve (*c*) in figure 1*d*.

The simulation, shown in the supplementary material, confirms that the excellent cloaking for the inclusion position of figure 1*c*, exemplified in figure 1*d*, holds irrespective of the inclusion position. It has thus been conclusively demonstrated that the cloaking is of sufficient quality to render the interference pattern almost immune to movement in the position of the cloaked obstacle. In particular, movement of the cloaked obstacle, it would seem, does not yield any information about the passage of waves through one slit or the other. This consideration would be important if one were able to carry out an experiment in which single quantized elements of vibration were in the system at any given instance in time. The quantum mechanical view would be that, if no path information were available from measurements, the interference fringes behind the double slit should persist.

This proposed quantum experiment raises interesting questions if an appropriate vibration transducer were embedded within the cloak, so that information about vibrations moulded by the cloak were available to experimentalists. One would assume, in line with the results of say optical experiments of the type referred to in [38], that any path information gained in this way would be evident in a change in the fringe pattern. This suggests the interest of a comprehensive quantum mechanical treatment of the interaction between mechanical cloaks and measurement systems.

## 4. Cloaking with a lattice

Cloaks designed using transformation optics may have such extreme physical attributes that the requisite materials cannot be physically realized without recourse to metamaterials. It is with this motivation in mind that the following approximate cloak in the low-frequency regime is developed. The cloak is constructed as an approximation to the continuum square cloak considered earlier, but is realized using a discrete lattice structure, formed from rods and point masses. The advantage of a discrete structure over a continuous material is that much higher contrasts in material properties are easily realizable using lattices. The development of an approximate cloaking material using a lattice may allow the practical construction of cloaks. In the following discussion, it is emphasized that repeated indices are not summed over.

**Figure 9.**
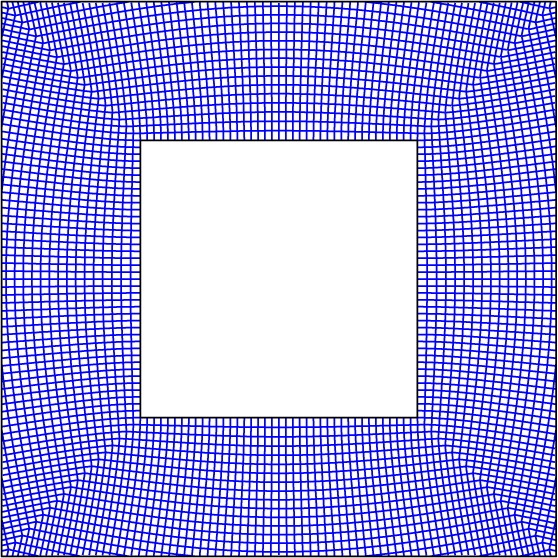
The lattice formed from the principal directions of the stiffness matrix for the continuum cloak. (Online version in colour.)

With reference to the formulae for the Jacobian of the transformation in §2*a*, the symmetric stiffness matrices ***C***^(*i*)^=[*μ*/*J*^(*i*)^]***J***^(*i*)^***J***^(*i*)^T^^ are positive-definite. Therefore, the stiffness matrix admits the following diagonalization:
4.1
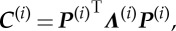
where 
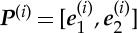
 are the matrices with columns consisting of the principal directions (eigenvectors) of ***C***^(*i*)^, and 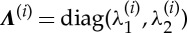 is the diagonal matrix of the corresponding ordered [positive] eigenvalues such that 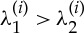. The eigenvectors yield the principal lattice vectors of the locally orthogonal lattice with homogenized stiffnesses 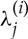 in direction 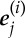. In particular, the lattice nodes lie at the intersection points of the solutions of the following nonlinear system of first-order differential equations:
4.2
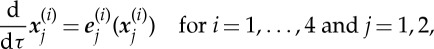
for some array of initial positions, where 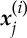 is the position vector along the characteristic defined by 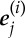 inside the *i*th side of the cloak and *τ* parametrizes the curve. Naturally, this would lead to a lattice with curved links. However, for a sufficiently refined lattice the curved members may be replaced with linear links. The lattice links are then the linearization of the characteristic between two neighbouring nodes on the characteristic. Figure 9 shows the geometry of the lattice formed from the principal vectors of the stiffness matrix. Requiring local conservation of flux allows the stiffness of the lattice link parallel to 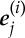 to be determined as 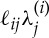, where ℓ_*ij*_ is the length of the link along 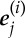. The distribution of nodal mass may be determined by evaluating the integral

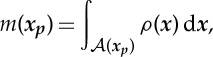
over the unit cell 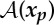 containing the lattice node at ***x***_***p***_.

In principle, the lattice cloak may be constructed exactly as described above and illustrated in figure 9. However, for narrow cloaks where *w*/*a*≪1, the locally orthogonal lattice depicted in figure 9 may be approximated by a globally orthogonal regular square lattice. A regular square lattice is more convenient to implement compared with the non-globally orthogonal lattice generated from the eigendecomposition of the stiffness matrix. Although the geometry of the approximate lattice is regular, it should be emphasized that the stiffness of the links and mass of the nodes vary with position according to the projection of ***A***(***x***) and *ρ*(***x***) as described above.

### (a) Geometry and governing equations for an inclusion cloaked by a lattice

**Figure 10.**
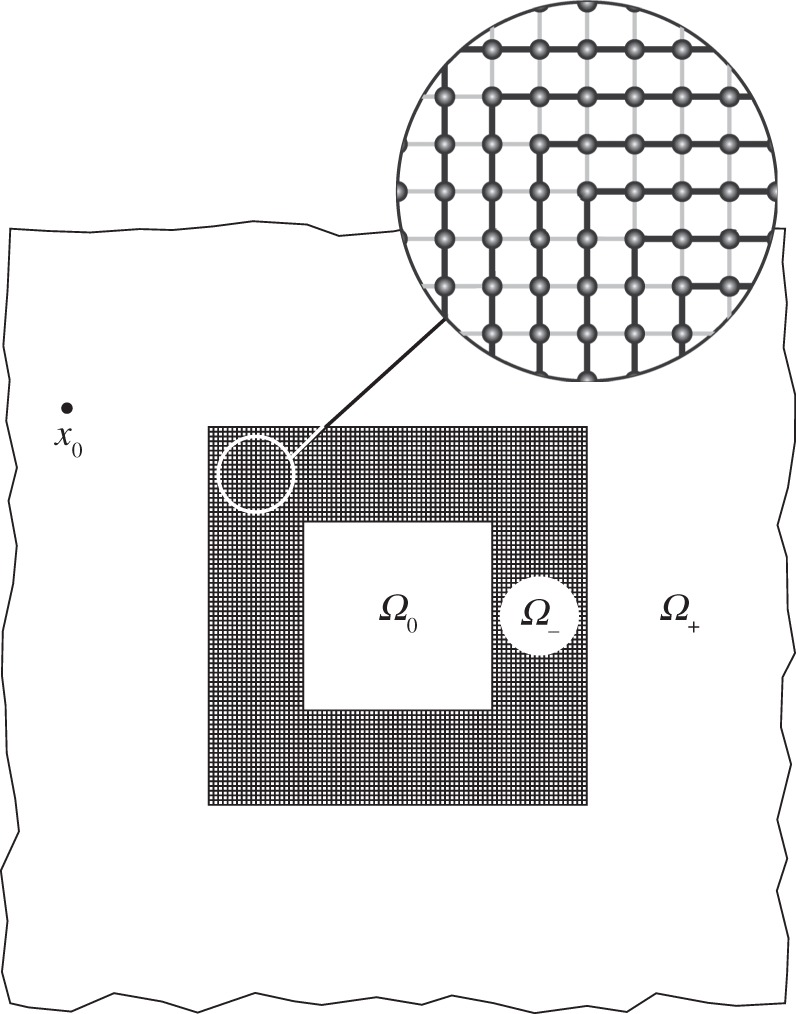
The lattice cloak *Ω*_−_, surrounding the square inclusion *Ω*_0_, embedded in the ambient medium *Ω*_+_. The thick black lines in the lattice cloak indicate links of high stiffness or conductivity, while the thick grey lines indicate links of low or stiffness conductivity.

Consider a square inclusion *Ω*_0_={***x***:|*x*_1_|<*a*,|*x*_2_|<*a*}, *a*>0, embedded in 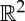, surrounded by a cloak *Ω*_−_={***x***:*a*<|*x*_1_|<*a*+*w*,*a*<|*x*_2_|<*a*+*w*}\*Ω*_0_, where *w*>0 is the thickness of the cloak. The cloak consists of a discrete lattice structure with lattice points at ***x***=ℓ***p***, where 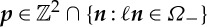. The lattice is statically anisotropic with links parallel and perpendicular to the boundaries having contrasting material properties, as shown in figure 10.

As for the continuum cloak, solutions of the Helmholtz equation are of primary interest. In particular, the following problem for the field *u*(***x***) is studied
4.3
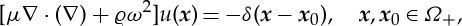

4.4
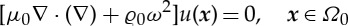

4.5
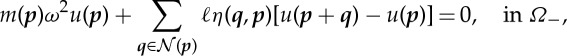
where ***e***_*i*_=[*δ*_*i*1_,*δ*_*i*2_]^T^, 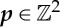, and 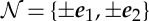 is the set of nearest neighbours. The stiffness and density of the ambient continuum are denoted by *μ* and *ϱ* respectively, whilst the corresponding quantities of the inclusion are denoted by *μ*_0_ and *ϱ*_0_. The stiffness of the lattice links are the restriction of the eigenvalues of the stiffness matrix to the links. In particular, for the link connecting nodes ***p*** and ***p***+***q***, *η*(***q***,***p***) takes the value 
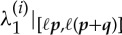
 if the vector ***q*** is parallel to the exterior boundary of the cloak, *Γ*^(*i*)^, and 
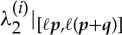
 otherwise. The corner regions are matched as illustrated in figure 10. Here, 
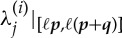
 indicates the restriction of 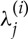 to the line [ℓ***p***,ℓ(***p***+***q***)]. The associated interface conditions corresponding to continuity of tractions are
4.6
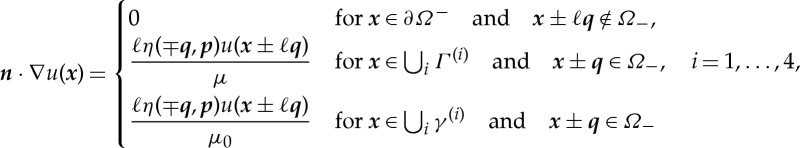
and the Sommerfeld radiation condition at infinity. The quantity *η*(***q***,***p***) is the projection of the diagonalized stiffness matrix onto the lattice link connecting lattice points ***p*** and ***p***+***q***.

Physically, (4.3)–(4.6) correspond to the problem of the propagation of time-harmonic waves of angular frequency *ω* generated by a point load at ***x***_0_. The field *u*(***x***) then corresponds to the out-of-plane displacement amplitude. The region *Ω*_−_ consists of an array of nodes of mass *m*, connected by massless rods of length ℓ and stiffness according to their orientation.

### (b) Illustrative lattice simulations

The approximate lattice cloaks were examined using the finite element software Comsol Multiphysics. Perfectly matched layers were used in the vicinity of the boundary of the computational domain in order to simulate an infinite domain. For the purpose of illustration, a square of semi-width *a*=0.5, surrounded by a lattice cloak with *w*=0.1 and links of length 5×10^−3^ was used. The inclusion is located at the origin of the computational window.

#### (i) A basic lattice cloak

Before proceeding to the illustrative simulations for the regular lattice with heterogeneous distributions of stiffness and mass, it is instructive to consider a simple approximation. Many cloaks created from transformation optics have the general characteristic of having a high-phase speed parallel to the boundary of the cloak, and a low-phase speed in the direction normal to the boundary [39]. Therefore, as an initial approximation, the case of a regular square lattice with a homogeneous, but orthotropic distribution of stiffness and a homogeneous distribution of mass is considered. Consider the right-hand side of the cloak *Ω*^(1)^_−_. For a narrow cloak with *w*/*a*≪1, *x*_1_∼*a*+*w* and hence the density may be approximated by *ρ*∼1+*a*/*w*. The greatest contrast in stiffness occurs at *x*_2_=0, thus the vertical links are assigned stiffness 
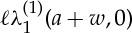
 and the horizontal links stiffness 
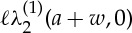
. The mass of the nodes is ℓ^2^(1+*a*/*w*). The material properties of the remaining three sides of the cloak are adjusted accordingly.

**Figure 11.**
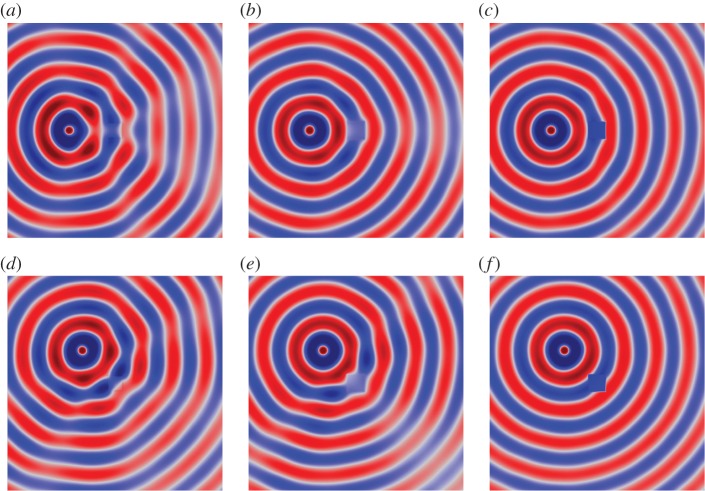
Plots of the field *u*(*x*) for a cylindrical wave incident on a square inclusion in the absence of a cloak (*a*,*d*), a square inclusion coated with the *basic* lattice (*b*,*e*), and an inclusion coating with the refined lattice (*c*,*f*). Here, the angular frequency of excitation is *ω*=3 and the source is located at *x*_0_=[−3,0]^T^ in (*a*–*c*), and at 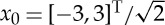 in (*e*–*f*). The colour scale is as indicated in figure 5. (*a*) Uncloaked, (*b*) basic cloak, (*c*) refined cloak, (*d*) uncloaked, (*e*) basic cloak and (*f*) refined cloak. (Online version in colour.)

**Figure 12.**
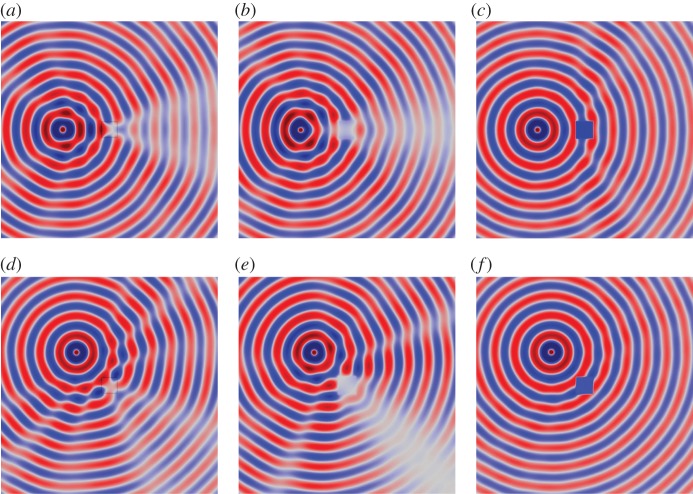
Plots of the field *u*(*x*) for a cylindrical wave incident on a square inclusion in the absence of a cloak (*a*,*d*), a square inclusion coated with the *basic lattice model* (*b*,*e*), and an inclusion coating with the refined lattice (*c*,*f*). Here, the angular frequency of excitation is *ω*=10 and the source is located at *x*_0_=[−3,0]^T^ in (*a*–*c*), and at 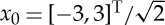 in (*e*–*f*). The colour scale is as indicated in figure 5. (*a*) Uncloaked, (*b*) basic cloak, (*c*) refined cloak, (*d*) uncloaked, (*e*) basic cloak and (*f*) refined cloak. (Online version in colour.)

Figures 11 and 12 show the field *u*(***x***) for the uncloaked inclusion (*a*) and (*d*), and the inclusion cloaked with this *basic* cloak (*b*) and (*e*). For *ω*=3, figure 11 indicates that the *basic* cloak partially mitigates the shadow cast by the inclusion and acts to reform the cylindrical wavefronts behind the inclusion. As illustrated in figure 12, this partial cloaking effect deteriorates with increasing frequency. Indeed, in some cases, the presence of the lattice cloak seems to increase the shadow region. Table 3 details the values of the scattering measures for the fields illustrated in figures 11 and 12. The scattering measures shown in table 3 suggest that, although visually the basic lattice cloak appears to work reasonably well, this may not be the case. The fact that the basic lattice cloak increases the scattering measure compared with the uncloaked inclusion further emphasizes the need for an objective measure of the quality of cloaks, rather than simply relying on visual observations.

**Table 3.** The scattering measures corresponding to the simulations for the *basic lattice model* shown in figures 11 and 12.

source		scattering measure 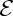		
position	frequency	uncloaked	cloaked	*Q*
*scattering region 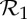 *				
[−3,0]^T^	3	0.1430	0.1662	0.1617
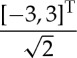	3	0.1113	0.1816	0.6327
[−3,0]^T^	5	0.1529	0.2495	0.6318
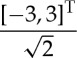	5	0.2002	0.3538	0.7676
*scattering region 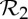 *				
[−3,0]^T^	3	0.2341	0.3362	0.4363
[−3,0]^T^	5	0.3224	0.4671	0.4489
*scattering region 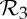 *				
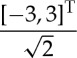	3	0.1578	0.3455	1.189
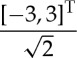	5	0.2988	0.6011	1.012

This increase in the scattering measure by the basic lattice cloak motivates the introduction of the following refined model.

#### (ii) A refined lattice cloak

Consider now the lattice described in §4*a*, i.e. the regular square lattice with inhomogeneous distribution of stiffness and mass. Figures 11 and 12 show the field *u*(***x***) for the uncloaked inclusion and the inclusion with a lattice cloaking. With reference to the simulations for the *basic* cloak (*b*) and (*e*) the *refined* lattice cloak (*c*) and (*f*), it is observed that the efficiency of the *refined* lattice cloak, while not as high as that of the continuum cloak, is much greater than that of the *basic* cloak. The table of scattering measures for the approximate cloak is shown in table 4 and further evidences the effectiveness of the *refined* lattice cloak. Indeed, for several simulations (in particular those where the scattering measure is taken over the forward or corner scattering regions 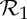 and 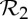, respectively) the efficiency of the *refined* cloak in reducing the scattering measure approaches that of the continuum cloak.

**Table 4.** The scattering measures corresponding to the simulations shown for the *refined lattice model* in figures 11 and 12.

source		scattering measure 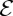		
position	frequency	uncloaked	cloaked	*Q*
*Scattering region 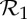 *				
[−3,0]^T^	3	0.1430	0.01191	0.8929
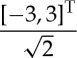	3	0.1113	3.385 × 10^−3^	0.9763
[−3,0]^T^	5	0.1529	0.04324	0.7173
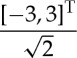	5	0.2002	0.03125	0.8438
*scattering region 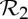 *				
[−3,0]^T^	3	0.2341	0.01150	0.9508
[−3,0]^T^	5	0.3224	0.0172	0.9508
*scattering region 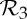 *				
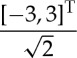	3	0.1578	5.047 × 10^−3^	0.9680
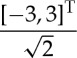	5	0.2988	0.02114	0.9292

As expected the effectiveness of the lattice cloaks reduce with increasing frequency. However, for sufficiently low frequencies the *refined* lattice cloak in particular, works well.

## 5. Concluding remarks

This work represents a comprehensive treatment of a non-singular cloak for a square inclusion. The significant advantage of this continuous cloak is the straightforward correspondence with a discrete metamaterial lattice structure. Such a connection may present a method through which a physical cloak may be fabricated. The material and geometric properties of the discrete cloak are directly linked to the properties of the continuum cloak, and, hence, to the properties of the formal map. The effectiveness of such discrete cloaks, particularly at low frequencies, was demonstrated through numerical simulations and the use of objective scattering measures.

Particular attention was paid to the objective measurement of the quality of the cloaking effect. The quality of the cloaks was primarily assessed using a scattering measure introduced as an *L*_2_ norm of the difference between the cloaked field and the ideal unperturbed field. A further demonstration of the efficacy of the square *push out* cloak was presented via the classical Young's double slit experiment. It was shown that the interference pattern on the observation screen was significantly modified when an obstacle was place in front of one of the apertures. However, if the obstacle was cloaked, then the interference pattern remained almost entirely unperturbed. This numerical experiment presents a further, perhaps more interesting, method through which the quality of particular cloaks may be examined. Moreover, the experiment raises interesting questions regarding the interaction between cloaking and quantum mechanics.

## Footnotes

1 Throughout this paper, all numerical parameters are normalized such that *μ*=*ϱ*=1 unless otherwise stated.
